# Quercetin Protects against Obesity-Induced Skeletal Muscle Inflammation and Atrophy

**DOI:** 10.1155/2014/834294

**Published:** 2014-12-28

**Authors:** Ngoc Hoan Le, Chu-Sook Kim, Taesun Park, Jung Han Yoon Park, Mi-Kyung Sung, Dong Gun Lee, Sun-Myung Hong, Suck-Young Choe, Tsuyoshi Goto, Teruo Kawada, Rina Yu

**Affiliations:** ^1^Department of Food Science and Nutrition, University of Ulsan, Ulsan 680-749, Republic of Korea; ^2^Department of Food and Nutrition, Yonsei University, Seoul 120-749, Republic of Korea; ^3^Department of Food Science and Nutrition and Research Institute for Bioscience & Biotechnology, Hallym University, Chuncheon 200-702, Republic of Korea; ^4^Sookmyung Women's University, Seoul 140-742, Republic of Korea; ^5^School of Life Science and Biotechnology, Kyungpook National University, Daegu 702-701, Republic of Korea; ^6^Graduate School of Agriculture, Kyoto University, Uji, Kyoto 611-0011, Japan

## Abstract

Skeletal muscle inflammation and atrophy are closely associated with metabolic impairment such as insulin resistance. Quercetin, a natural polyphenol flavonoid, is known to elicit anti-inflammatory and antioxidant activities. In this study, we investigated its effect on obesity-induced skeletal muscle inflammation and atrophy in mice. Male C57BL/6 mice were fed a regular diet, a high-fat diet (HFD), and an HFD supplemented with quercetin for nine weeks. Quercetin reduced levels of inflammatory cytokines and macrophage accumulation in the skeletal muscle of the HFD-fed obese mice. It also reduced transcript and protein levels of the specific atrophic factors, Atrogin-1 and MuRF1, in the skeletal muscle of the HFD-fed obese mice, and protected against the reduction of muscle mass and muscle fiber size. In vitro, quercetin markedly diminished transcript levels of inflammatory receptors and activation of their signaling molecules (ERK, p38 MAPK, and NF-*κ*B) in cocultured myotubes/macrophages, and this was accompanied by reduced expression of the atrophic factors. Together, these findings suggest that quercetin reduces obesity-induced skeletal muscle atrophy by inhibiting inflammatory receptors and their signaling pathway. Quercetin may be useful for preventing obesity-induced muscle inflammation and sarcopenia.

## 1. Introduction

Skeletal muscle is the most abundant tissue, with a wide variety of physiological functions, and thus muscle loss results not only in physical dysfunction but also in metabolic impairment [1]. Inflammatory mediators such as tumor necrosis factor alpha (TNF*α*) and interleukin-6 (IL-6) are important mediators of catabolic responses such as protein loss and of metabolic disturbances such as insulin resistance [2]. They promote protein degradation by upregulating the expression of two muscle-specific ubiquitin E3-ligases, F-box protein (MAFbx/Atrogin-1), and muscle ring-finger protein 1 (MuRF1), which are involved in the ubiquitin proteasome pathway, and so lead to skeletal muscle atrophy [3]. Recent studies have shown that obesity is associated with skeletal muscle loss and dysfunction, referred to as sarcopenic obesity [4]. It is likely that obesity-induced inflammation, which is characterized by increases in macrophage infiltration and in levels of inflammatory cytokines/chemokines (e.g., TNF*α*; monocyte chemoattractant protein 1, MCP-1) [5–7], is associated with this muscle atrophy. Given that muscle atrophy and inflammation exacerbate obesity-induced insulin resistance [8], dietary components that suppress obesity-induced skeletal muscle inflammation and/or atrophy could be useful for preventing obesity-related metabolic disorders.

Quercetin (3,3′,4′,5,7-pentahydroxyflavone) is a polyphenolic flavonoid compound found in many dietary sources and intensively investigated for its multiple health-related effects such as antioxidant and anti-inflammatory activities [9–12]. We previously showed that quercetin inhibits the release of chemokines such as macrophage inflammatory protein-1*α* (MIP-1*α*/CCL3) from cocultured adipocytes/macrophages by interfering with inflammatory signaling [13] and also reduced adipose inflammation by inhibiting macrophage accumulation and cytokine release in obese adipose tissue [14]. Interestingly, this flavonoid also inhibits oxidative stress-induced and/or unloading-derived disused skeletal muscle atrophy in both skeletal muscle cells and muscle tissue [15, 16]. However, it is not clear whether it can prevent obesity-related skeletal muscle inflammation and atrophy.

In the present study, we for the first time demonstrate that quercetin diminishes skeletal muscle atrophy through reducing obesity-induced muscle inflammation and that it does so by inhibiting inflammatory receptor signaling. Quercetin may be useful for protecting against obesity-induced sarcopenia and sarcopenia-related metabolic dysregulation.

## 2. Materials and Methods

### 2.1. Animals

Eight-week-old male C57BL/6 mice were individually housed in a specific pathogen-free animal facility, with a 12-12 h light-dark cycle. The mice were fed a regular diet (RD) (13% of calories as fat from soybean oil; Harlan Teklad, Madison, WI, USA), a high-fat diet (HFD) (60% of calories as fat from lard and soybean oil; Research Diets Inc., New Brunswick, NJ, USA), or an HFD supplemented with 0.05% quercetin (0.05 g quercetin/100 g diet) (HFD/Q0.05) or 0.1% quercetin (HFD/Q0.1) for 9 weeks. Quercetin was purchased from Jena Bioscience GmbH (Jena, Germany). The animals were given free access to food and water. All animal experiments were approved by the animal ethics committee of the University of Ulsan and conformed to National Institutes of Health guidelines. The animals were killed by CO_2_ asphyxiation, and their muscles were dissected.

### 2.2. Cell Culture and Treatment

The mouse myoblast cell line C2C12 and the monocyte/macrophage-like cell line Raw264.7 were purchased from the American Type Culture Collection (ATCC, Manassas, USA). The C2C12 myoblasts (5 × 10^5^ cells/mL) were grown at 37°C in 5% CO_2_ in Dulbecco's modified Eagle's medium (DMEM) (Gibco, NY, USA) containing 10% fetal bovine serum (FBS) (Gibco), 100 units/mL penicillin, 100 *μ*g/mL streptomycin (Invitrogen, Carlsbad, CA, USA), and 20 *μ*g/mL gentamicin (Gibco). When the cells reached 100% confluence, the medium was replaced with differentiation medium that consisted of DMEM plus 2% horse serum (Gibco), which was changed after 2 days. Raw264.7 cells were cultured to 80% confluence at 37°C in 5% CO_2_ in RPMI medium (Gibco) containing 10% FBS, 100 units/mL penicillin, 100 *μ*g/mL streptomycin (Invitrogen), and 20 *μ*g/mL gentamicin (Gibco). For coculture, the Raw264.7 cells were detached with 0.05% trypsin-EDTA (Gibco) and Raw264.7 cells corresponding to 25% of the number of confluent myoblasts (8–10 × 10^5^ myoblasts/well of 24-well plate) were directly seeded into culture plates containing 3 day-differentiated myotubes in serum-free DMEM. The myotube and Raw264.7 cells were cocultured (Co). As the control, the same numbers of Raw264.7 cells and myotubes were cultured separately and mixed after being collected/harvested, named as mixture (Mix), same as FFA treated condition, which was named as Mix/FFA. The cocultures were incubated for 24 h, following treatment with 500 *μ*M palmitic acid (FFA) in serum-free DMEM containing 50 *μ*M BSA (Co/FFA). Palmitic acid (Sigma) was dissolved in ethanol and conjugated with BSA at a 10 : 1 molar ratio before use. The established myotubes and macrophages were pretreated with 50 *μ*M quercetin in serum-free DMEM for 1 h (Co/FFA/Q). An equal amount of DMSO was used as a control for the quercetin-treated group. After incubation, the culture supernatants were collected to measure cytokine concentrations by ELISAs; at the same time samples of the cells were washed twice with PBS and lysed in Trizol reagent (Invitrogen) for quantitative real time PCR and in lysis buffer for Western blot analysis.

### 2.3. Quantitative Real Time PCR

Gastrocnemius muscle tissues were collected and stored at −20°C in RNAlater (Ambion, Austin, TX, USA). Total RNA was extracted from 50 mg muscle tissue samples, or cells were lysed with Trizol reagent (Invitrogen). Two microgram aliquots of total RNA were reverse transcribed using M-MLV reverse transcriptase (Promega, Madison, WI, USA). qRT-PCR amplification of the cDNA was performed in duplicate with a SYBR premix Ex Taq kit (TaKaRa Bio Inc., Forster, CA, USA) using a Thermal Cycler Dice (TaKaRa Bio Inc., Japan). All reactions were performed according to the same schedule: 95°C for 10 s followed by 40 cycles of 95°C for 5 s and 60°C for 30 s. Results were analyzed with Real Time System TP800 software (Takara Bio Inc.) and all values were normalized to the levels of the house-keeping gene *β*-actin. The primers used in the analysis are listed in Table 1.

### 2.4. Measurement of Cytokine Proteins

Gastrocnemius muscles (100 mg) were homogenized in 1 mL of 100 mM Tris-HCl and 250 mM sucrose buffer (pH 7.4) supplemented with 0.25% protease inhibitor cocktail (Sigma) and centrifuged at 10,000 g for 10 min at 4°C. Levels of TNF*α* and MCP-1 in the tissue homogenates or in the culture supernatants were measured by enzyme-linked immunosorbent assays (ELISAs) using the OptEIA mouse TNF*α* and/or MCP-1 sets (BD Biosciences, NJ, USA). Amounts of cytokine were adjusted for the protein content of the homogenates determined with a BCA protein assay kit (Pierce, Rockford, IL, USA).

### 2.5. Western Blotting

Mice were killed by CO_2_ asphyxiation. Briefly, gastrocnemius muscle tissues were dissected and immediately frozen in liquid nitrogen. For protein extraction, tissues and cell cultures were homogenized in lysis buffer containing 150 mM NaCl, 50 mM Tris-HCl, 1 mM EDTA, 50 mM NaF, 10 mM Na_4_P_2_O_7_, 1% IGEPAL, 2 mM Na_3_VO_4_, 0.25% protease inhibitor cocktail, and 1% phosphatase inhibitor cocktail (Sigma). The homogenates were centrifuged at 12000 g for 20 min at 4°C. Samples of 50 *μ*g and 10 *μ*g total protein extracted from tissues and cell cultures, respectively, were subjected to Western blot analysis using polyclonal antibodies to CD68, MuRF1, nuclear factor of kappa light polypeptide gene enhancer in B-cells inhibitor alpha (I*κ*B*α*, Santa Cruz Biotechnology, Santa Cruz, CA, USA), and phosphorylated extracellular signal-regulated kinase (p-ERK1/2), ERK1/2, phosphorylated p38 mitogen-activated protein kinase (p-p38 MAPK), and p38 MAPK (Cell Signaling, Danvers, MA, USA). *α*-tubulin was used as a loading control, measured with mouse *α*-tubulin antibody (Abcam, MA, USA).

### 2.6. Histological Analysis

Gastrocnemius muscles were fixed overnight at room temperature in 10% formaldehyde and embedded in paraffin. Eight-micron-thick sections were stained with hematoxylin-eosin (H&E), mounted on glass slides, and observed with an Axio-Star Plus microscope. The diameters of the muscle fibers were determined using microscope AxioVision software (Carl Zeiss, Gottingen, Germany). Five microscopic fields for each sample were counted at 200x magnification.

### 2.7. Statistical Analysis

The results are presented as means ± standard error of the mean (SEM). Variables were compared using Student's *t*-test or analysis of variance (ANOVA) with Duncan's multiple-range test. Differences were considered significant at *P* < 0.05.

## 3. Results

### 3.1. Effect of Quercetin on Inflammatory Responses in the Skeletal Muscle of HFD-Fed Mice

**Figure 1:**
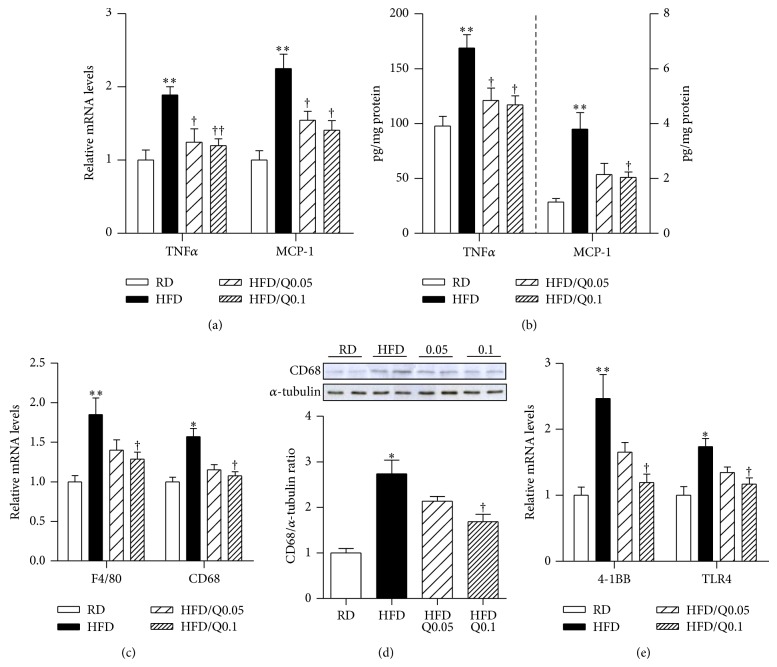
Effect of quercetin on obesity-induced inflammatory responses in skeletal muscle. Eight-week-old male C57BL/6 mice were fed a regular diet (RD), a high-fat diet (HFD), or an HFD supplemented with 0.05% quercetin (HFD/Q0.05) or 0.1% quercetin (HFD/Q0.1) for 9 weeks. ((a), (c), (e)) qRT-PCR analysis of cytokine transcripts (TNFα and MCP-1), macrophage markers (F4/80 and CD68), and inflammatory receptors (4-1BB and TLR4) in gastrocnemius muscle. mRNA levels were normalized to the levels of β-actin gene. (b) Protein levels of cytokines TNFα, MCP-1 measured by ELISA. (d) CD68 and α-tubulin were determined by Western blotting. The intensities of the bands were measured densitometrically using ImageJ software. Data are means ± SEM of 6 animals per group. ^*^ *P* < 0.05 and ^**^ *P* < 0.01 between the HFD and RD groups; ^†^ *P* < 0.05 and ^††^ *P* < 0.01 between the HFD/Q0.05, HFD/Q0.1 groups and the HFD group.

We first examined the effects of quercetin on skeletal muscle inflammation. As shown in Figures 1(a) and 1(b), HFD-fed mice had increased levels of inflammatory cytokines such as TNF*α* and MCP-1 compared with RD-fed mice, and exposure to quercetin reduced these effects. Skeletal muscle transcript and protein levels of F4/80 and CD68, which were higher in the HFD-fed mice than in the RD-fed mice, were also reduced by quercetin in a dose-dependent manner (Figures 1(c) and 1(d)). Subsequently, we examined the effect of quercetin on inflammatory receptors such as TNF receptor superfamily member 9 (4-1BB) and toll-like receptor 4 (TLR4), which are known to promote obesity-induced inflammatory responses in skeletal muscle [17, 18]. Transcript levels of the inflammatory receptors were lower in the skeletal muscle of the HFD/Q0.05- and HFD/Q0.1-fed mice than in that of the HFD-fed mice (Figure 1(e)).

### 3.2. Effect of Quercetin on Obesity-Induced Skeletal Muscle Atrophy

**Figure 2:**
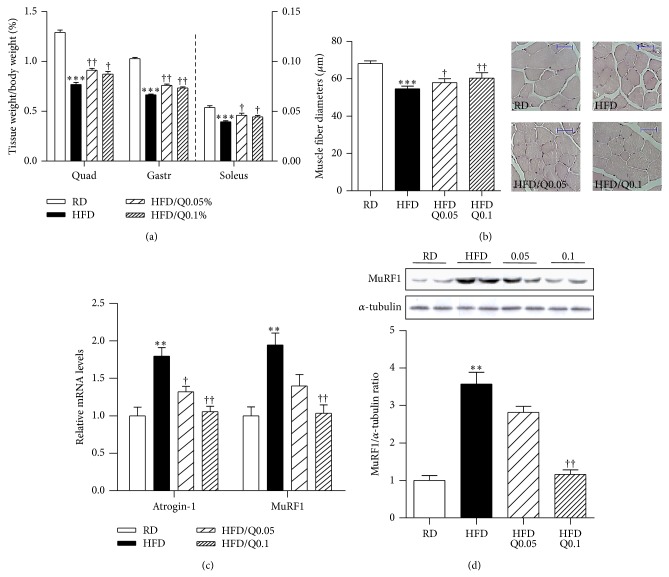
Effect of quercetin on obesity-induced skeletal muscle atrophy. (a) Ratios of quadriceps (Quad), gastrocnemius (Gastr), and soleus muscle weights to whole body weight. (b) Mean diameters of gastrocnemius muscle fibers. The diameters were determined with microscope AxioVision software. Five microscopic fields per sample were counted at 200x magnification. (c) qRT-PCR analysis of the expression of atrophic genes Atrogin-1 and MuRF1 in gastrocnemius muscle. Relative mRNA levels were normalized to levels of the β-actin gene. (d) MuRF1 and α-tubulin were determined by Western blotting. The intensities of the bands of MuRF1 and α-tubulin were measured with ImageJ software. Data are means ± SEM of 6 animals per group. ^**^ *P* < 0.01 and ^***^ *P* < 0.001 between the HFD and RD groups; ^†^ *P* < 0.05 and ^††^ *P* < 0.01 between the HFD/Q0.05, HFD/Q0.1, and HFD groups.

Skeletal muscle atrophy is characterized by reduced muscle mass and fiber size [16, 19]. Quercetin did not alter food intake, and the body weight of HFD/Q0.05-fed mice had a tendency to gain less weight than the HFD-fed mice, as previously reported [14], but not different from the HFD/Q0.1-fed mice (Table 2). As shown in Figure 2(a), the ratio of skeletal muscle weight to whole body weight was lower in HFD-fed than in RD-fed mice, and this reduction was prevented in the HFD/Q0.05- and HFD/Q0.1-fed mice. Similarly, histological examination of gastrocnemius cross sections showed that mean muscle fiber diameter was higher in HFD/Q0.05- and HFD/Q0.1-fed mice than in HFD-fed mice (Figure 2(b)). Next we examined the effect of quercetin on the expression of atrophic factors. As shown in Figure 2(c), expression of the atrophic genes Atrogin-1 and MuRF1 was upregulated in the HFD-fed mice, but not in the HFD/Q0.05- and HFD/Q0.1-fed mice. The level of MuRF1 protein was also significantly reduced in the HFD/Q0.1-fed mice compared to the HFD-fed mice and was similar to the level in the RD-fed mice (Figure 2(d)).

### 3.3. Effect of Quercetin on the Inflammatory Response and Expression of Atrophic Factors in Cocultured Myotubes/Macrophages Treated with Palmitic Acid

**Figure 3:**
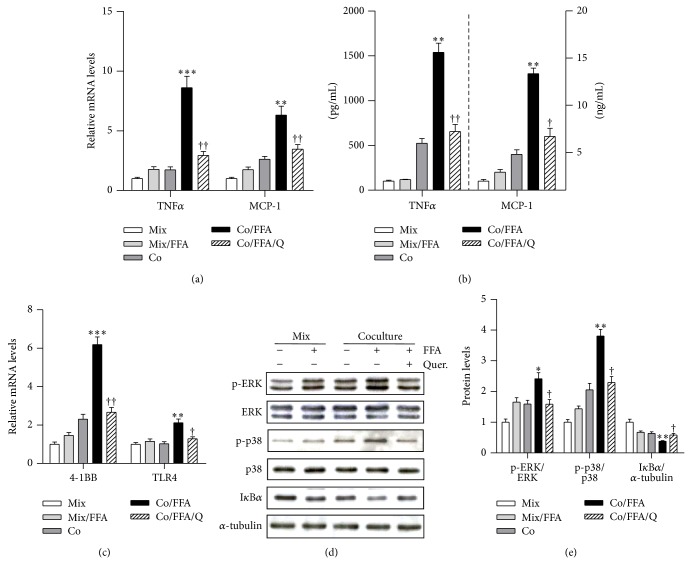
Quercetin suppresses inflammatory responses in cocultured myotubes and macrophages treated with FFA. C2C12 myotubes (Mb) were cocultured with Raw264.7 macrophages (M⌀) for 24 h (Co) and then treated with 500 μM palmitic acid for the next 24 h (Co/FFA). As the control, the same numbers of Mb and M⌀ were cultured separately and mixed after being collected/harvested, named as mixture (Mix), same as FFA treated condition, which was named as Mix/FFA. The established Mb and M⌀ were pretreated with 50 μM quercetin in serum-free DMEM for 1 h (Co/FFA/Q). ((a), (c)) qRT-PCR analysis of transcripts of inflammatory cytokines (TNFα and MCP-1) and inflammatory receptors (4-1BB and TLR4). (b) Protein levels of TNFα and MCP-1 released into the culture medium were measured by ELISA. (d) Phosphorylated ERK, ERK, phosphorylated p38 MAPK, p38 MAPK, IκBα, and α-tubulin were determined by Western blotting. (e) The intensities of the bands were measured using ImageJ. Data are means ± SEM of three independent triplicate experiments. ^*^ *P* < 0.05, ^**^ *P* < 0.01, and ^***^ *P* < 0.001 for the differences between the Co/FFA group and the Mix, Mix/FFA, or Co groups. ^†^ *P* < 0.05 and ^††^ *P* < 0.01 for the differences between the Co/FFA/Q and Co/FFA groups.

**Figure 4:**
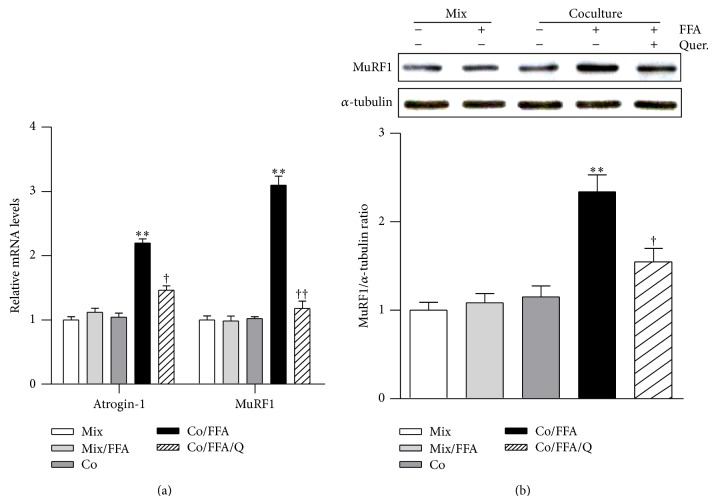
Quercetin suppresses the expression of atrophic factors in cocultured myotubes and macrophages treated with FFA. C2C12 Mb were cocultured with M⌀ for 24 h and then treated with 500 μM FFA for the next 24 h. Quercetin (50 μM) was added for 1 h before coculture started, and DMSO was used as control. (a) qRT-PCR analysis of transcripts of atrophic genes, including Atrogin-1 and MuRF1. (b) Expressions of MuRF1 and α-tubulin were determined by Western blotting. The intensities of the bands were measured using ImageJ software. Data are means ± SEM of three independent triplicate experiments. ^**^ *P* < 0.01 for the differences between the Co/FFA group and the Mix, Mix/FFA, or Co groups. ^†^ *P* < 0.05 and ^††^ *P* < 0.01 for the differences between the Co/FFA/Q and Co/FFA groups.

Because both macrophages and free fatty acids increase in obese skeletal muscle [20, 21], we cocultured C2C12 skeletal muscle myotubes (Mb) with Raw264.7 macrophages (MQ) in the presence of palmitic acid (FFA) to mimic the obese microenvironment and treated the cells with quercetin. Quercetin significantly inhibited the increases in transcript and protein levels of inflammatory cytokines TNF*α* and MCP-1 in these cocultures (Figures 3(a) and 3(b)). Transcripts of other cell surface molecules that are involved in inflammatory responses, including 4-1BB and TLR4, were markedly reduced by quercetin (Figure 3(c)). In agreement with this, coculture of Mb/MQ in the presence of FFA induced phosphorylation of ERK and p38 MAPK and degradation of I*κ*B*α*, while the activation of these inflammatory signaling molecules was significantly inhibited by the presence of quercetin (Figures 3(d) and 3(e)). Moreover, coculture of Mb/MQ along with FFA strongly increased the expression of the atrophic genes Atrogin-1 and MuRF1, and as in the case of obese skeletal muscle (Figure 2(c)), this increase was inhibited by quercetin (Figure 4(a)). Quercetin was also found to reduce the level of MuRF1 protein in cocultured Mb/MQ treated with FFA (Figure 4(b)).

## 4. Discussion

Skeletal muscle inflammation plays a crucial role in muscle atrophy and in systemic metabolic dysfunction [5, 22]. It is thought that a 10% increase in the ratio of skeletal muscle mass to total body weight is associated with 11% reduction in the risk of insulin resistance [23]. Hence, the prevention of skeletal muscle inflammation and/or atrophy has been predicted to improve obesity-induced metabolic dysregulation [19]. Studies have shown that quercetin increases skeletal muscle mitochondria function and attenuates insulin resistance in obese mice [24, 25]. It also decreases circulating markers of inflammation in mice fed a high-fat diet [26] and inflammatory response in skeletal muscle of genetically obese mice [27]. However, it remains unclear whether quercetin protects obesity-related skeletal muscle inflammation and atrophy.

We first examined the effect of quercetin on obesity-induced skeletal muscle inflammation. Quercetin decreased levels of inflammatory cytokines/chemokines (TNF*α* and MCP-1) in the skeletal muscle of HFD-fed obese mice, and this inhibitory effect was confirmed in cocultured muscle cells/macrophages treated with FFA. Given that quercetin lowers the plasma concentration of FFA in genetically obese Zuker rats [28], the reduction of inflammatory cytokines by quercetin may be partly associated with reduction of circulating FFA. Next, we inquired whether the reduction of skeletal muscle inflammation by quercetin protected against obesity-related skeletal muscle atrophy. Atrogin-1 and MuRF1 are believed to be key players in the development of skeletal muscle atrophy by promoting the degradation of proteins [3]. Functional studies have shown that ablation of Atrogin-1 or MuRF1 protects mice from muscle atrophy, while overexpression of Atrogin-1 reduces myotube cell size [29, 30]. Intriguingly, we found that quercetin supplementation prevented obesity-induced decreases in muscle mass and fiber size in the skeletal muscle of the HFD-fed obese mice. In line with this, expression of atrophic factors Atrogin-1 and MuRF1 was downregulated, indicating that quercetin protects against obesity-induced skeletal muscle atrophy, presumably through inhibition of muscle inflammation.

It should be noted that transcription of the protein ligases Atrogin-1 and MuRF1 is regulated by signaling pathways such as ERK, p38 MAPK, and I*κ*B kinase (IKK)/nuclear factor kappa-light-chain-enhancer of activated B cell (NF-*κ*B) [20, 31–35]. Hence, the protective effect of quercetin may occur through inhibition of the phosphorylation of these kinases and presumably of NF-*κ*B activation. Indeed, we found that the activation of inflammatory signaling molecules was significantly inhibited by the presence of quercetin, indicating that the protective effect of quercetin against muscle atrophy is mediated by inhibition of inflammatory signaling and cytokine release. Like obesity-induced adipose inflammation, skeletal muscle inflammation is characterized by infiltration of macrophages and their interaction with muscle cells, and this plays a crucial role in the increased production of inflammatory cytokines [7, 20]. We confirmed that quercetin supplementation markedly reduced transcript and protein levels of the macrophage-specific markers CD68 and F4/80 in the skeletal muscle of HFD-fed obese mice, indicating that it prevented macrophage accumulation, which is in line with our in vitro observation that inhibits the chemotactic activity of macrophages [13]. Thus, the reduced macrophage infiltration by quercetin may decrease the cross-talk between macrophages/muscle cells, which promotes their inflammatory responses [7, 17], and this may be favorable for the reduction of skeletal muscle inflammation.

Inflammatory receptors such as toll-like receptors (TLRs) and the TNF receptor superfamily (TNFRSF) participate in initiating obesity-induced inflammatory responses. For example, binding of FFAs to TLR4 leads to downstream signaling that activates inflammatory signaling molecules such as the MAP kinases ERK and p38 [36, 37]. Meanwhile, engagement of 4-1BB (TNFRSF9) with its ligand 4-1BBL (TNRSF9) can activate the inflammatory signaling pathway involving MAP kinases in T cells and macrophages [38], and we have also shown that the 4-1BB/4-1BBL interaction on adipocytes/macrophages or muscle cells/macrophages promotes obesity-induced adipose and muscle inflammation [17, 39]; hence inflammatory receptor expression and their signaling are considered useful targets for protecting against obesity-induced inflammation [17, 39]. Interestingly, we observed that quercetin reduced transcripts of the inflammatory receptors TLR4 and 4-1BB in skeletal muscle, as well as in Mb/MQ cocultures. Moreover, in the cocultures it also inhibited phosphorylation of ERK and p38 MAPK and the degradation of I*κ*B*α*, and these are downstream components of the signaling pathways activated by inflammatory receptors [17, 39, 40]. These findings indicate that the reduction of inflammatory cytokine production in obese skeletal muscle by quercetin may be at least in part attributed to downregulation of inflammatory receptors* per se* and inhibition of their inflammatory signaling.

**Figure 5:**
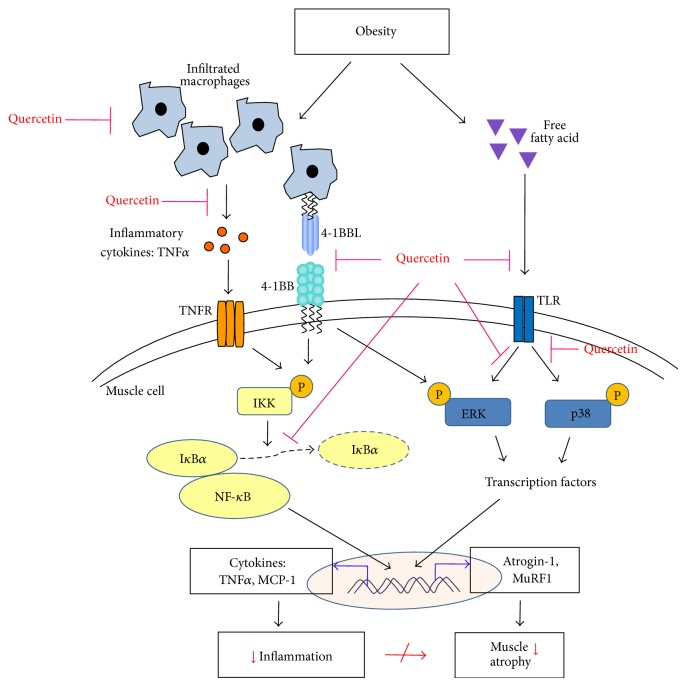
Schematic representation of the effect of quercetin on obesity-induced skeletal muscle inflammation and muscle atrophy. Obesity leads to infiltration of macrophages and increases FFA in skeletal muscle. Macrophages release inflammatory cytokines and/or directly contact muscle cells, leading to activation of inflammatory signaling pathways, which are also induced by binding of FFA to TLRs. The inflammatory signaling pathway induces expression of atrogenes. This leads to loss of muscle mass, and these changes ultimately contribute to local and systemic insulin resistance. Quercetin inhibits macrophage infiltration and activation of inflammatory pathways, leading to diminished expression of atrophic genes.

In conclusion, the present study for the first time demonstrates that quercetin can prevent obesity-induced skeletal muscle atrophy by suppressing inflammatory responses. These effects are associated with downregulation of the inflammatory receptors TLR4 and 4-1BB and inhibition of their inflammatory signaling pathways (Figure 5). Quercetin may be a useful dietary component for preventing obesity-induced muscle inflammation and sarcopenia.

## Figures and Tables

**Table 1:** Mouse primers used for qRT-PCR.

Gene	Forward primer (5′ → 3′)	Reverse primer (5′ → 3′)
TNF*α*	AAGCCTGTAGCCCACGTCGTA	GGCACCACTAGTTGGTTGTCTTTG
MCP-1	GCATCCACGTGTTGGCTCA	CTCCAGCCTACTCATTGGGATCA
4-1BB	CTCTGTGCTCAAATGGATCAGGAA	TGTGGACATCGGCAGCTACAA
TLR4	GGGCCTAAACCCAGTCTGTTTG	GCCCGGTAAGGTCCATGCTA
Atrogin-1	ACATTCTGCCAGCTGCTGTTTC	TGAGTTGGATGCTGGGCCTAC
MuRF1	TGTCTCACGTGTGAGGTGCCTA	CACCAGCATGGAGATGCAGTTAC
*β*-actin	CATCCGTAAAGACCTCTATGCCAAC	ATGGAGCCACCGATCCACA

**Table 2:** Body weight, food intake, and organ weight.

Groups	RD	HFD	HFD/Q0.05	HFD/Q0.1
Initial weight (g)	20.99 ± 0.40	20.91 ± 0.37	20.71 ± 0.33	20.79 ± 0.33
Final weight (g)	27.63 ± 0.49	41.78 ± 1.56^**^	37.75 ± 1.48	39.33 ± 1.66

Food intake (g/day)	4.15 ± 0.05	3.15 ± 0.08^*^	3.13 ± 0.08	3.27 ± 0.08
Energy intake (Kcal/d)	13.28 ± 0.15	16.48 ± 0.41^*^	16.37 ± 0.42	17.19 ± 0.45

Epididymal fat (g)	0.22 ± 0.05	2.62 ± 0.15^**^	2.02 ± 0.29^†^	2.26 ± 0.13
Total muscle weight (mg)	655.42 ± 7.76	608.26 ± 9.17^**^	648.55 ± 10.25^†^	648.82 ± 14.62^†^
Quadriceps (mg)	353.53 ± 6.46	317.58 ± 8.10^**^	344.35 ± 6.80^†^	342.91 ± 10.67
Gastrocnemius (mg)	284.05 ± 3.04	274.42 ± 3.78	286.88 ± 4.78	288.48 ± 4.55^†^
Soleus (mg)	14.84 ± 0.54	16.26 ± 0.47	17.31 ± 0.83	17.43 ± 0.59

Eight-week-old male C57BL/6 mice were fed a regular diet (RD), a high-fat diet (HFD), or an HFD supplemented with 0.05% quercetin (HFD/Q0.05) or 0.1% quercetin (HFD/Q0.1) for 9 weeks. Data are means ± SEM of 6 animals per group. ^*^ *P* < 0.05 and ^**^ *P* < 0.01 between the HFD and RD groups; ^†^ *P* < 0.05 between the HFD/Q0.05, HFD/Q0.1 groups and the HFD group.
